# Change in Astigmatism After Temporal Clear Corneal Cataract Extraction in the Pediatric Population

**DOI:** 10.2174/1874364100802010043

**Published:** 2008-03-18

**Authors:** Helene Y Lam, Kimberly G Yen

**Affiliations:** Department of Ophthalmology, Cullen Eye Institute, Baylor College of Medicine, Houston, TX, USA

## Abstract

**Purpose:**

To evaluate the early postoperative change in astigmatism in pediatric patients having cataract extraction with intraocular lens implantation using sutured temporal clear corneal incision. Methods: A retrospective chart review was performed on all pediatric patients who underwent clear corneal cataract surgery with intraocular lens implantation between 12/01/2005 and 11/30/2006. Results: A total of 31 eyes of 22 patients who underwent temporal clear corneal cataract surgery and intraocular lens implantation were included. The mean patient age at surgery was 6.05 years (range 1.5 months to 17 years). Mean postoperative refractive astigmatism the first day after surgery was 2.35±1.37 diopters (D). There was a statistically significant decrease in mean postoperative astigmatism by postoperative week 1 to 1.45 ± 0.79 D. Mean astigmatism was 1.48 ± 0.98 D by postoperative months 2-4, which was not a statistically significant change from postoperative day 1. Conclusions: Postoperative astigmatism can be expected after sutured temporal clear corneal surgery in pediatric patients and decreases over time without removal of sutures. The amount of postoperative astigmatism in children requires close follow-up after pediatric cataract surgery.

## INTRODUCTION

Intraocular lens (IOL) implantation at the time of cataract surgery is currently a widely accepted method of treatment for pediatric patients with cataracts [1]. As the techniques for pediatric cataract surgery have evolved, small incision temporal clear corneal incisions are also being used frequently in pediatric patients who are receiving intraocular lenses [1]. Because the pediatric eyes are much softer than the eyes of adults and since pediatric patients may be more prone to trauma and less compliant, pediatric clear corneal wounds are usually sutured.

Since amblyopia is a concern in children, the amount of postoperative astigmatism is an important factor to consider. Early postoperative astigmatism is of concern, since amblyopia treatment is usually initiated as soon as possible after cataract surgery and precise optical correction of astigmatism is needed to maximize visual improvement. In this study, we evaluate the early postoperative changes in refractive astigmatism after cataract extraction in the pediatric population using sutured clear corneal temporal wounds

## METHODS

After approval by the Baylor College of Medicine Institutional Review Board, a retrospective chart review was performed on all pediatric patients under the age of 18 who had cataract surgery with intraocular lens implantation performed by one surgeon (KGY) between 12/01/2005 and 11/30/2006. Patients were excluded if they had corneal abnormalities (such as a corneal scar) or if a retinoscopic refraction could not be performed due to retinal pathology. Data collected included the etiology of the cataract, age at surgery, type of cataract, preoperative retinoscopic refraction (if it could be obtained), and postoperative retinoscopic refraction at each follow up visit.

Intraocular lens calculations were performed using immersion A-scan ultrasonography for axial length measurements and keratometry using a handheld keratometer. In cases in which the posterior capsule was to be left intact, a temporal clear corneal incision was made using a 3.2 mm keratome. A continuous curvilinear capsulorrhexis was performed with utrata forceps and aspiration of the lens was accomplished using an automated irrigation/aspiration handpiece with the Accurus system (Alcon Laboratories). In patients in which a primary posterior capsulotomy and anterior vitrectomy was planned, the anterior capsulotomy and lens aspiration were performed using a mechanical vitrectomy handpiece through a temporal incision created with a 20-gauge MVR blade. This incision was measured with calipers and enlarged to 3.2mm with the MVR blade prior to insertion of the intraocular lens. A primary posterior capsulotomy and anterior vitrectomy was then performed in a standardized fashion through the pars plana approach [2].

All patients received a single piece, foldable acrylic IOL (Acrysof, SN60AT, Alcon) that had an optic diameter of 6.0 mm and a total diameter of 13.0mm. The optic and haptics were placed in the capsular bag. The corneal wounds were then closed with 910 polyglactin sutures, with three interrupted sutures placed in the temporal clear-corneal incision, and one suture placed in each paracentesis.

All patients were treated postoperatively with prednisolone acetate 1% eye drops every 2 hours for the first week followed by slow tapering over the next 4 weeks. Topical moxifloxacin was given 4 times a day for 1 week postoperatively. No sutures were removed in any of the patients.

Follow up visits were performed at one day, one week, 1 month, and 4 months after surgery. The refractive error of the surgically treated eye was measured at each visit using manual retinoscopy by a pediatric ophthalmologist. No sutures were removed during follow-up. Statistical comparisons of outcome variable were performed using the Students’ paired t test.

## RESULTS

A total of 35 eyes of 26 patients underwent clear corneal temporal cataract surgery with intraocular lens implantation during the study period. Two patients had traumatic cataracts with associated corneal wounds and were excluded from analysis; two patients had retinal abnormalities that prevented them from being refracted and were also excluded from analysis. A total of 31 eyes of 22 patients met inclusion criteria and were included for analysis. The mean patient age at surgery was 6.05 years (range 1.5 months to 17 years). A total of 65% of the cataracts were congenital and 35% were acquired. Table **1** shows the patient demographics and etiology of the cataracts.

Mean preoperative refractive astigmatism was 0.87 ± 1.00 diopters (D). Preoperative refractive astigmatism could not be measured by retinoscopy in 13 patients due to density of the cataract. Mean postoperative refractive astigmatism first day after surgery was 2.35 ±1.37 (D). The mean astigmatism was 1.45 ± 0.79 (D) at postoperative week 1. At postoperative month 1, the mean refractive astigmatism was 1.60 ± 1.06 (D). The decrease in refractive astigmatism from postoperative day 1 to postoperative week 1 was statistically significant (p=0.017), but the difference from postoperative week 1 to postoperative month 1 was not significant (p=0.058). At postoperative months 2-4, the mean refractive astigmatism was 1.48 ± 0.98 (D), which was not a statistically significant change from postoperative day 1 (p=0.261) or postoperative month 1 (p=0.115) (Table **2**).

The rate of with-the-rule (WTR) and against-the-rule (ATR) astigmatism was evaluated. The type of astigmatism was classified into 3 subgroups. Astigmatism in the meridian between 60 degrees and 120 degrees was defined as WTR and in the meridian between 1 degree and 30 degrees and 150 degrees and 180 degrees as ATR. All other astigmatism was classified as oblique. Preoperatively, 23% of eyes had WTR astigmatism, 8% had ATR astigmatism, and 3% had oblique astigmatism. Twenty six percent were astigmatically neutral with the rest of eyes unable to be measured. At postoperative day 1, 29% had WTR astigmatism, 25% had ATR astigmatism, and 23% had oblique astigmatism. By posteropative day 1, there was a shift to WTR astigmatism with 55% of eyes in this category and by postoperative month 1, 74% of eyes had WTR astigmatism.

## CONCLUSIONS

In this study, we evaluated the early change in postoperative refractive astigmatism in pediatric patients after cataract extraction with IOL implantation through a 3.2mm temporal clear corneal incision closed with dissolvable suture. Our results demonstrate that early postoperative astigmatism is induced in pediatric patients after a sutured temporal clear corneal wound. This astigmatism decreases initially and then appears to stabilize during the first 2-4 months after surgery without suture removal with the most change occurring in the first postoperative week.

Postoperative astigmatism is especially important in children due to the adverse effect astigmatism can have on children’s visual development and the treatment of amblyopia. Previous studies have demonstrated that most pediatric ophthalmologists prefer to prescribe glasses at a threshold of 1.50 D difference in astigmatism between the two eyes in children greater than age two [3]. Other studies have suggested that as low as 1.00 D amount of anisometropic astigmatism can be amblyogenic in pediatric patients [4]. In our patients, average postoperative refractive astigmatism was 2.35D at postoperative day 1 decreasing to an average of 1.48D by postoperative months 2 to 4. Because the amount of astigmatism declines in the postoperative period, we feel that early postoperative astigmatism should be corrected in patients and the amount of astigmatism reassessed in the first few months after surgery.

With the advent of small-incision (< 4.0mm) clear corneal cataract surgery in the adult population, postoperative astigmatism can be significantly reduced because of the self-sealing corneal wound which often requires no sutures [5]. Cataract surgery in children can also be performed through small clear-corneal incisions, however the increased scleral elasticity of the pediatric eye predisposes the patients to a higher incidence of wound leak and gaping [6]. Furthermore, pediatric patients tend to be more prone to eye rubbing and less compliant with activity restrictions. Therefore, the pediatric wounds are usually sutured. Absorbable suture is preferred to avoid a return trip to the operating room to remove the sutures. The suturing of the corneal wounds in an eye of higher scleral elasticity may be one reason for the increase in astigmatism postoperatively. Our preference is to use polyglactin 910. Because the half-life of polyglactin 910 sutures in terms of tensile strength is 2 weeks [7], change in astigmatism can be expected in the early postoperative period.

In the present study, postoperative astigmatism is highest on the first postoperative day. Our results are comparable to the study by Spierer and Bar-Sela which showed that in clear-corneal cataract incisions, postoperative astigmatism declines significantly by 3 months postoperatively, and stabilizes by 3 to 5 months postoperatively [8,9].

Clear-corneal incisions appear to have less induced astigmatism than scleral tunnel incisions in pediatric patients. Prior studies using scleral tunnel incisions have shown early post-operative astigmatism as high as 14.00D [10]. Brown, *et al.* used a 6.25mm scleral-tunnel incision and reported early postoperative astigmatism of up to 8.00D [11]. Our data is comparable to the clear corneal incision data from the study by Bradfield *et al.*, which showed a postoperative astigmatism range of 0-4.50D the first month after surgery [12] however in that study a superior clear corneal incision was used. Some studies have demonstrated that temporal incisions induce less astigmatism than superior wounds [13,14] but others have shown comparable results [14].

Our patients also had a shift toward WTR astigmatism over time. Previous studies have demonstrated that flattening of the cornea occurs along the incisional meridian [14]. This results in WTR astigmatic changes when a temporal incision is used [14,15], consistent with the results of our study.

It has been demonstrated that patients younger than 36 months of age have a lower amount of postoperative astigmatism [12]. This has been attributed to the increased elasticity of the pediatric corneas, which may allow the eye to regain normal curvature more easily. Due to the low number of patients under 36 months in our study, this comparison could not be performed.

As with any retrospective study, there are several limitations to our study. Preoperative refractive astigmatism could not be measured in all patients due to density of the cataracts. Additionally, several patients were not seen at their designated follow-up visits or presented after a longer or shorter interval. Even though all patients were scheduled for follow up examinations at regular postoperative intervals, some patients did not return for follow up at the designated times. Preoperative and postoperative keratometry, if it could be measured, would be a useful method to compare postoperative astigmatism to preoperative values. However, this data is not available since the vast majority of our patients would not be cooperative for keratometry in the clinic. Finally, our study has a limited number of patients. This may have limited statistically significance at some time points.

In conclusion, postoperative refractive astigmatism in pediatric patients can be expected to occur after sutured temporal clear corneal cataract surgery with IOL placement. This refractive astigmatism decreases early after surgery and seems to stabilize by the first 2 to 4 months after surgery. Because the amount of astigmatism may be higher in the early postoperative period, it requires close follow-up after surgery. Consideration should be given to reassessing refraction in the first few months after surgery to ensure optimal correction of refractive error in patients in the amblyopic age range. Further studies including larger numbers of children of younger age, especially those less than 36 months of age, are warranted to better clarify the types of astigmatic changes in the younger age group.

## Figures and Tables

**Table 1:** Demographics

Total number of patients included	22
Total number of eyes included	31
Average age at surgery	6 years
Range of ages	1.5 months to 17 years
Lamellar cataract	7
Hereditary cataract	11
Posterior lenticonus	2
Steroid induced	7
Radiation induced	2
Traumatic	1
ROP related	1

**Table 2:** Mean Posteroperative Refractive Astigmatism

Time	Eyes	Mean Refractive Astigmatism (D)±SE	Range (D)
Pre-op	18	0.87±1.00	0-2.50
Post-op day 1	24	2.35±1.37	0.50-4.50
Post-op week 1	23	1.45±0.79	0.0-2.75
Post-op month 1	25	1.60±1.06	0.0-3.50
Post-op month 2-4	14	1.48±0.98	0.0-3.50
